# Age and serum anti-Müllerian hormone levels as predictors of time to return of menses after chemotherapy

**DOI:** 10.1530/RAF-24-0046

**Published:** 2025-01-11

**Authors:** Madhavi-Priya Singh, Rashi Kalra, Franca Agresta, Alec Leos, Samith Minu Alwis, Alex Polyakov, Genia Rozen, Kate Stern

**Affiliations:** ^1^ Austin Health, Melbourne, Victoria, Australia; ^2^ Melbourne IVF, East Melbourne, Victoria, Australia; ^3^ Royal Women’s Hospital, Melbourne, Victoria, Australia; ^4^ The University of Melbourne, Melbourne, Victoria, Australia; ^5^ Genea, Melbourne, Victoria, Australia

**Keywords:** anti-Müllerian hormone, chemotherapy, fertility, menses, oncofertility

## Abstract

**Abstract:**

Chemotherapeutic agents result in the loss of growing follicles, which can manifest as amenorrhoea. Alkylating chemotherapy (AC) is known to be more gonadotoxic than non-alkylating chemotherapy (NAC). Anti-Müllerian hormone (AMH), an indirect marker of ovarian reserve, and age have been investigated as predictors of ovarian function after chemotherapy; however, little is known about the time to return of menses. This study aimed to assess how patient age and baseline serum AMH levels at cancer diagnosis affect the time to return of menses post-chemotherapy. This retrospective cohort study examined oncology patients (*n* = 67) who underwent chemotherapy and were treated through the Reproductive Services Unit of two institutions in Melbourne, Australia. Primary outcomes included the correlation between age and baseline AMH with time to return of menses after chemotherapy. Secondary outcomes include the change in AMH levels at 6- and 12-months post-completion of chemotherapy. Pairwise correlation of the pre-chemotherapy AMH level and time to return of menses demonstrated statistical significance (Spearman’s coefficient, *ρ* = −0.40) for patients who underwent AC. This analysis in breast cancer patients who underwent AC displayed a negative correlation but was not statistically significant. No association was found between age and time to return of menses for all cancer (NAC or AC) or breast cancer patients who underwent AC. Higher AMH levels prior to AC were associated with an earlier return of menses after chemotherapy. Age at the commencement of chemotherapy was not associated with return of menses. Further prospective research is required to assess post-chemotherapy recovery of AMH.

**Lay summary:**

Chemotherapy, used to treat cancer, is known to damage women’s ovaries, with certain types having a more toxic effect than others. This may result in a temporary loss of periods while undergoing chemotherapy. AMH is a hormone produced by the ovaries and gives an indication of their level of function. This study looks at whether an individual's AMH or age when beginning chemotherapy can predict the time before the resumption of periods after completing chemotherapy. This study found that for cancer patients who underwent the chemotherapy type known to be more toxic to ovaries, the higher their AMH level was before beginning chemotherapy, the more rapidly their periods would return after completing chemotherapy. Age was not found to accurately predict how rapidly periods would return after completing chemotherapy. This information can be used to inform patients before treatment of the chances of periods returning and, consequentially, pregnancy after the completion of their chemotherapy.

## Introduction

Chemotherapeutic agents cause DNA damage and stop both malignant and non-malignant cells in mitosis (Helleday 2017). As a result, they target rapidly dividing cell types. One such target is growing ovarian follicles. The ‘follicular burn-out hypothesis’ (Roness *et al.* 2013) describes the loss of ovarian granulosa cells, resulting in a higher rate of recruitment and consequential atresia of primordial follicles. The potential consequences of chemotherapy, such as infertility, are becoming increasingly relevant with improved survival rates of oncology patients and older age at childbirth (Anderson & Cameron 2011). Between 2013 and 2017, the five-year survival rate of Australian females aged 20–39 diagnosed with breast cancer was 89.6% (Australian Institute of Health & Welfare, 2023, accessed 15 May 2023).

Anti-Müllerian hormone (AMH), as an indirect indicator of the total primordial follicle pool, is a valuable marker of ovarian reserve with a role in inhibiting the growth of primordial follicles during folliculogenesis (Durlinger *et al.* 2002). AMH is preferred to other biomarkers of ovarian reserve as its levels do not vary significantly during normal menstrual cycles and are only marginally affected by exogenous hormones, such as hormonal contraceptive interventions (Iwase *et al.* 2016).

The damage to ovarian reserve by chemotherapy can manifest as temporary or permanent amenorrhoea (Anderson *et al.* 2017). AMH is usually suppressed during chemotherapy. However, partial or complete recovery of AMH, and therefore menstruation, is possible after chemotherapy (Anderson & Cameron 2011). Anderson and coworkers found that pre-chemotherapy AMH levels could be used as a long-term predictor of ovarian function post-chemotherapy (Anderson *et al.* 2013). In addition, Behringer and coworkers explored the relevance of age as a contributing factor to the loss of ovarian reserve during treatment (Behringer *et al.* 2013).

There are numerous studies assessing chemotherapy-related amenorrhoea (CRA). However, little is known about the duration and return of menses. Certain chemotherapeutic agents, for example alkylating agents, are known to be more gonadotoxic than others (Wong & Anderson 2018) and are likely to have an influence on AMH and return of menses.

Prediction of recovery of AMH levels and the time to return of menses in these patients would aid in counselling, provision of patient information and fertility preservation strategies, resulting in better management of girls and women prior to gonadotoxic treatment. In addition, better knowledge about the resumption of menses after chemotherapy would allow for considerations of unplanned pregnancy and the need for contraception after chemotherapy, the commencement time for neoadjuvant hormone treatments in breast cancer and the ability to perform post-chemotherapy oocyte retrieval in the case of poor retrieval prior to chemotherapy.

Our primary objective was to assess how factors such as patient age and pre-chemotherapy AMH levels at cancer diagnosis affect the time to return of menses post-chemotherapy. Secondary outcomes that were evaluated include the change in AMH levels at 6- and 12-months post-completion of chemotherapy.

## Materials and methods

### Participants

A total of 1408 patients were reviewed by the Fertility Preservation Service and Reproductive Services Unit (RSU) of two institutions in Melbourne, Australia, between January 2012 and September 2020. Ethics approval was obtained from the Human Research Ethics Committee of Melbourne IVF (reference 70/19).

Of these patients, 307 were oncology patients, either receiving treatment or on review for fertility preservation.

Patients who were receiving chemotherapy for their cancer treatment were included (*n* = 280).

For each patient, baseline characteristics were retrieved from electronic patient files or paper files from the Medical Records department. Characteristics recorded included cancer type, cancer treatment type (chemotherapy, radiation, hormonal treatment), chemotherapy type, pre-chemotherapy AMH level, use of adjuvant hormonal therapy, treatment completion and return of menstruation. Where documentation in the initial clinical records was incomplete, clarification was obtained via phone follow-up.

Patients who underwent pelvic irradiation were excluded (*n* = 11) due to the radiation-induced permanent ovarian function loss and uterine damage (Kim *et al.* 2021). Patients taking goserelin for hormonal treatment of their cancer were also excluded (*n* = 11), as were patients below the reproductive age of 12 (*n* = 1).

**Figure 1:**
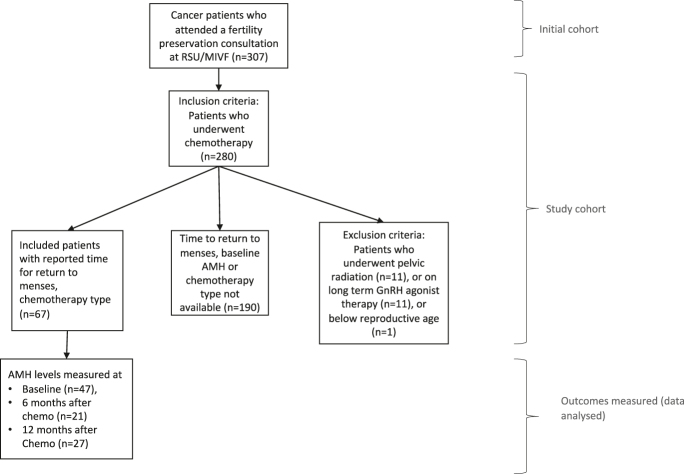
Study design.

For the remaining patients, 67 patients had a documented chemotherapy type (non-alkylating and alkylating agents) and the time to return of menses. Follow-up AMH levels 6 and 12 months after the conclusion of therapy were also recorded for these patients when available. This study design is displayed in Fig. 1.

AMH was measured in pmol/L. The assay utilised was the electrochemiluminescence immunoassay used on the Roche Cobas e 801 immunoassay analyser. Retrospective analysis of the data was performed as detailed below.

### Statistical analysis

Descriptive statistics were used to analyse the characteristics of the patient population, including cancer type and chemotherapy type. Statistical analysis was performed using RStudio version 4.2.1 (https://posit.co/download/rstudio-desktop/). Continuous variables were summarised as means and standard deviation if normally distributed, or median and interquartile range (IQR) if not normally distributed. Subgroup analysis of non-alkylating and alkylating chemotherapy (AC) groups, as well as breast cancer-only groups, was carried out. Spearman’s correlation coefficient (rho) was utilised to assess the pairwise correlation between time to return of menses and both age and serum AMH levels. *P* < 0.05 was considered statistically significant.

## Results

### Patient demographics

The age of the cohort ranged from 18 to 40 years, with a median age of 31 (IQR = 7). Pre-chemotherapy AMH levels ranged from 1.00 to 63.80 pmol/L, with a median of 18.93 (IQR = 18.75). There were 46/67 (68.66%) patients who underwent AC, while 21/67 (31.34%) underwent non-alkylating chemotherapy (NAC).

**Figure 2:**
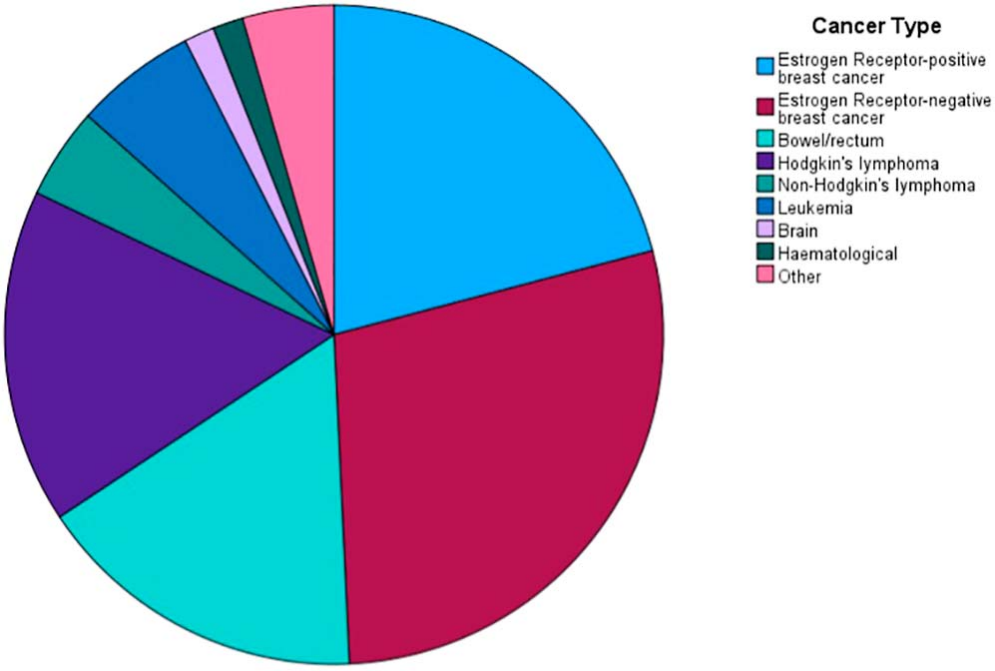
Distribution of cancer types.

The distribution of cancer types is demonstrated in Fig. 2. Fourteen of 67 (20.90%) patients had oestrogen receptor-positive (ER-positive) breast cancer, 19/67 (28.36%) had oestrogen receptor-negative (ER-negative) breast cancer, 11/67 (16.41%) had colorectal cancer, 11/67 (16.41%) had Hodgkin’s lymphoma, 3/67 (4.48%) had non-Hodgkin’s lymphoma, 4/67 (5.97%) had leukaemia, 1/67 (1.49%) had brain cancer, 1/67 (1.49%) had haematological malignancy, and 3/67 (4.48%) had other cancers.

The proportion of patients receiving goserelin during chemotherapy for ovarian protection was 53/67 (79.10%). Eight patients were not taking goserelin. Out of the patients who were not taking goserelin, 4/8 (50%) patients were diagnosed with ER-negative breast cancer, 1/8 (12.5%) with ER-positive breast cancer, 1/8 (12.5%) with lymphoma, and 2/8 (25.0%) with other cancer types.

Outcomes for all patients are shown in Table 1. Outcomes for breast cancer patients are displayed in Table 2.

**Table 1:** Outcomes measured for cancer patients.

	Mean ± SD	Median (IQR)	Range	*n*
Alkylating				
Age (years)	30.96 ± 5.57	32.00 (7.25)	18.00–40.00	46
Baseline AMH (pmol/L)	22.37 ± 10.37	24.20 (14.80)	5.10–47.70	32
AMH post-chemotherapy (pmol/L)				
6–12 months	7.99 ± 8.07	4.57 (13.29)	1.00–26.00	16
>12 months	10.07 ± 10.80	7.60 (8.25)	0.11–44.60	17
Time to return of menses (weeks)	16.70 ± 14.23	12.00 (16.00)	0.00–80.00	46
Goserelin				38
Non-alkylating				
Age (years)	29.52 ± 6.15	29.00 (9.00)	19.00–39.00	21
Baseline AMH (pmol/L)	17.00 ± 15.56	10.4 (15.30)	1.00–63.80	15
AMH post-chemotherapy (pmol/L)				
6–12 months	5.70 ± 8.11	3.00 (10.85)	0.00–20.00	5
>12 months	21.84 ± 14.31	24.55 (25.78)	0.40–43.80	10
Time to return of menses (weeks)	12.19 ± 10.79	8.00 (15.00)	0.00–44.00	21
Goserelin				15

**Table 2:** Outcomes measured for breast cancer patients.

	Mean ± SD	Median (IQR)	Range	*n*
Alkylating				
Age (years)	32.50 ± 3.94	32.00 (5.75)	23.00–39.00	28
Baseline AMH (pmol/L)	21.43 ± 11.22	23.40 (18.60)	5.10–47.70	23
AMH post-chemotherapy (pmol/L)				
6–12 months	6.90 ± 6.53	4.57 (9.46)	1.00–19.90	10
>12 months	8.44 ± 7.15	7.50 (8.30)	0.11–26.60	11
Time to return of menses (weeks)	17.64 ± 15.53	12.00 (16.00)	0.00–80.00	28
Goserelin				25
Non-alkylating				
Age (years)	33.80 ± 5.00	34.00 (9.50)	27.00–39.00	5
Baseline AMH (pmol/L)	24.96 ± 26.40	14.67 (44.96)	6.70–63.80	4
AMH post-chemotherapy (pmol/L)				
6–12 months	3.00*	3.00^†^	3.00–3.00	1
>12 months	26.93 ± 18.65	30.10^†^	6.90–43.80	3
Time to return of menses (weeks)	14.4 ± 17.57	8.00 (28.00)	0.00–44.00	5
Goserelin				2

* SD value is not available.

^†^ IQR value is not available.

### Correlation between time to return of menses and pre-chemotherapy AMH and age for all patients

**Figure 3:**
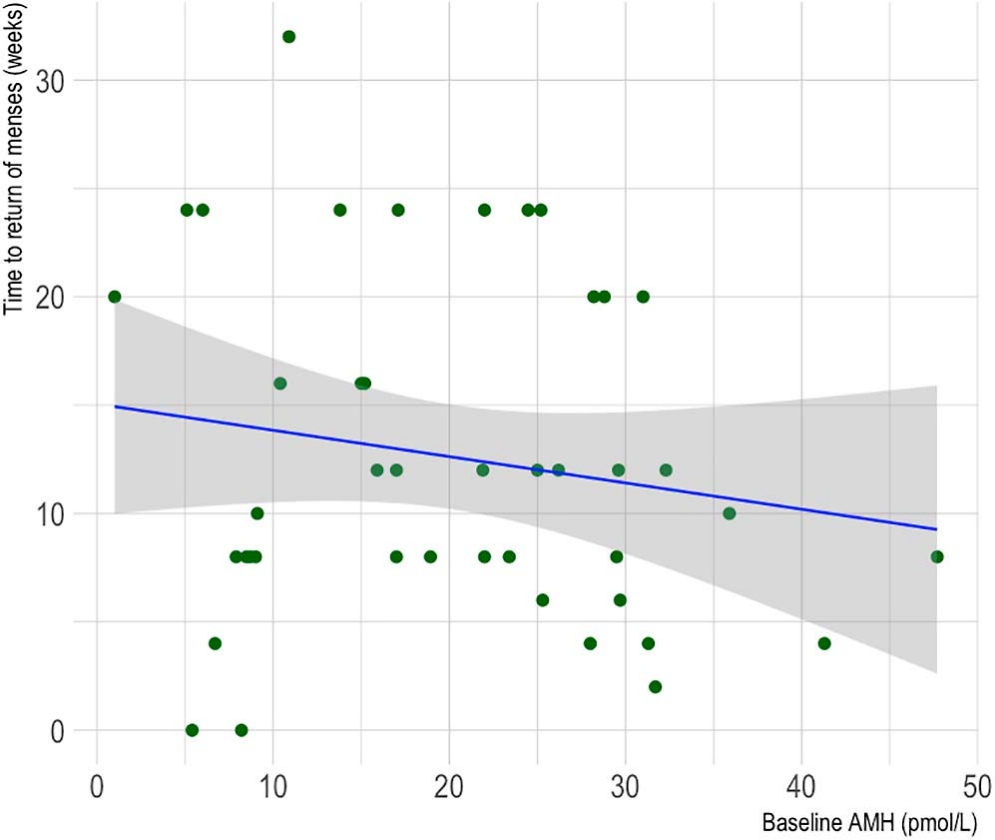
Scatter plot of the correlation between baseline serum AMH and time to return of menses for cancer patients. *R* = - 0.14, *P* = 0.36.

As shown in Fig. 3, no correlation was observed between baseline serum AMH and time to return of menses for all patients (Spearman’s rank correlation coefficient = −0.14, *P* = 0.36).

**Figure 4:**
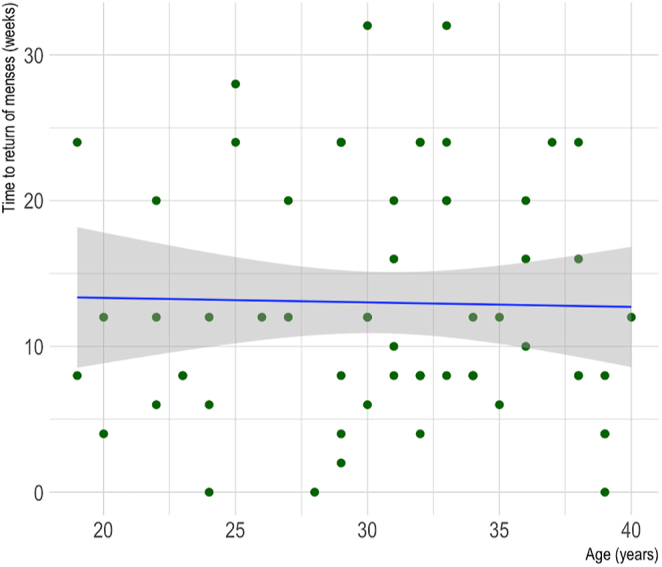
Scatter plot of the correlation between age and time to return of menses for cancer patients. *R* = −0.03, *P* = 0.83.

Figure 4 demonstrates that no correlation was observed between age and time to return of menses for all patients (Spearman’s rank correlation coefficient = −0.03, *P* = 0.83).

### Comparison of non-alkylating and alkylating chemotherapy groups

A one-sided Mann–Whitney U test was used to compare the distributions of time to return of menses for the AC and NAC groups. No difference was observed in the time to return of menses between the AC and NAC groups (AC mean 16.7 weeks, NAC mean 12.2 weeks, *W* = 376.5, *P* = 0.07).

### Correlation between time to return of menses and pre-chemotherapy AMH and age for AC patients

**Figure 5:**
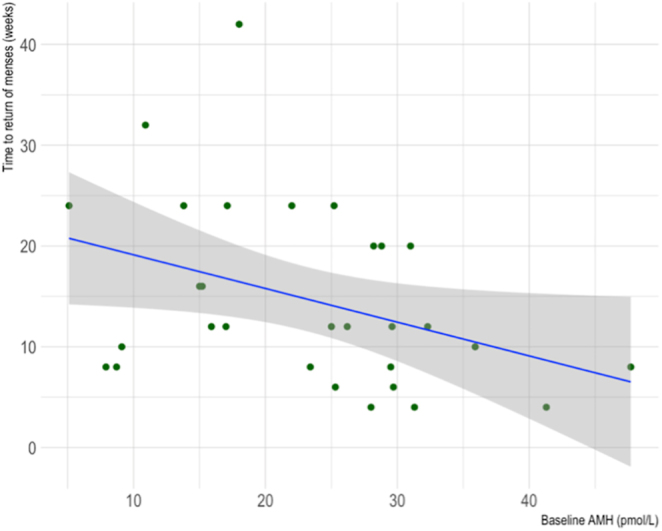
Scatter plot of the correlation between baseline serum AMH and time to return of menses for cancer patients who underwent AC. *R* = −0.40, *P* = 0.03.

As shown in Fig. 5, there was a negative correlation observed between baseline serum AMH and time to return of menses for AC patients (Spearman’s rank correlation coefficient = −0.40, *P* = 0.03).

**Figure 6:**
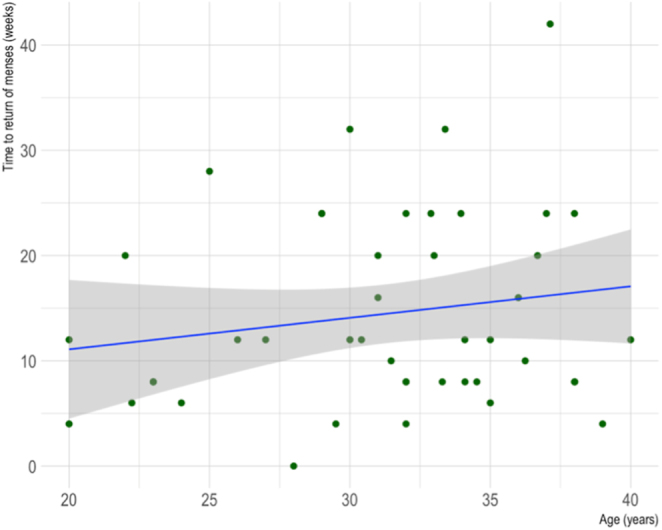
Scatter plot of the correlation between age and time to return of menses for cancer patients who underwent AC. *R* = 0.12, *P* = 0.44.

As observed in Fig. 6, no relationship was found between age and time to return of menses for AC patients (Spearman’s rank correlation coefficient = 0.12, *P* = 0.44).

### Correlation between time to return of menses and pre-chemotherapy AMH and age for NAC patients

**Figure 7:**
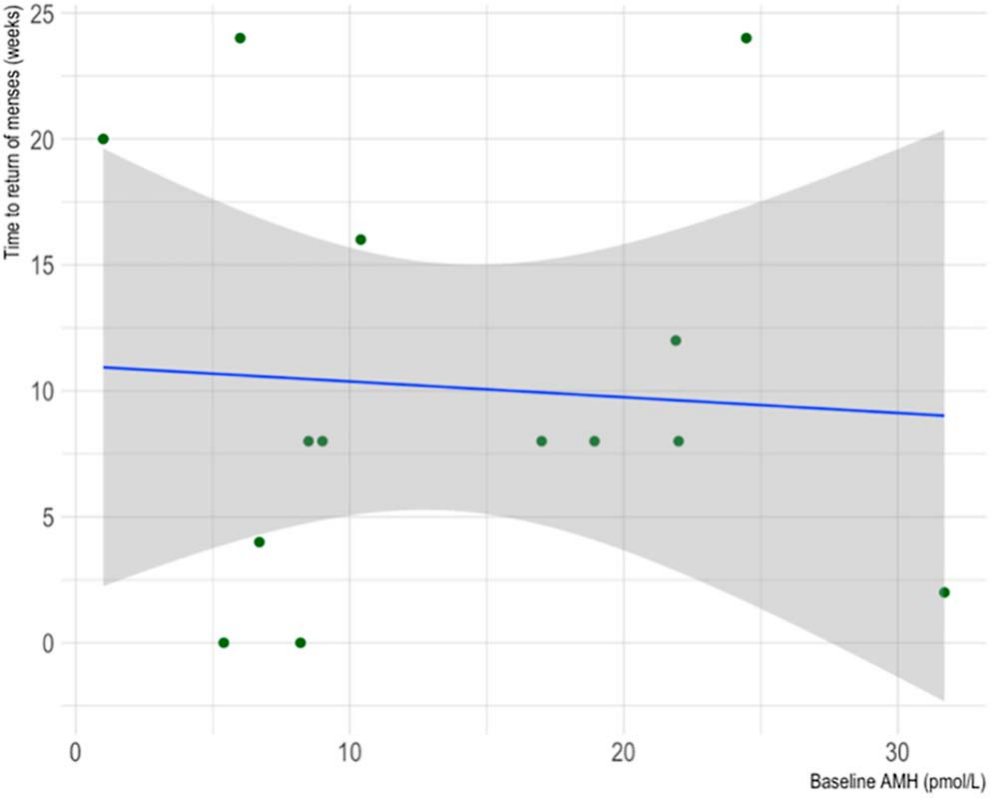
Scatter plot of the correlation between baseline serum AMH and time to return of menses for cancer patients who underwent NAC. *R* = −0.07, *P* = 0.82.

As shown in Fig. 7, no statistically significant association was observed between baseline serum AMH and time to return of menses for NAC patients (Spearman’s rank correlation coefficient = −0.07, *P* = 0.82).

**Figure 8:**
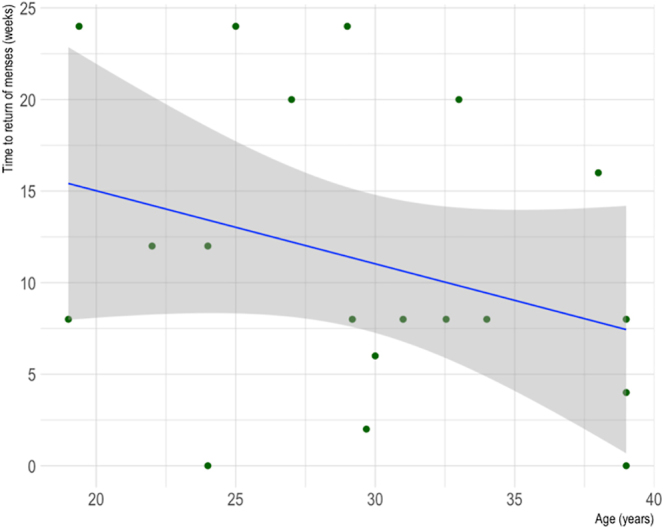
Scatter plot of the correlation between age and time to return of menses for cancer patients who underwent NAC. *R* = −0.36, *P* = 0.13.

As observed in Fig. 8, no statistically significant association was found between age and time to return of menses for NAC patients (Spearman’s rank correlation coefficient = −0.36, *P* = 0.13).

### Correlation between time to return of menses and pre-chemotherapy AMH and age for breast cancer patients who underwent AC

**Figure 9:**
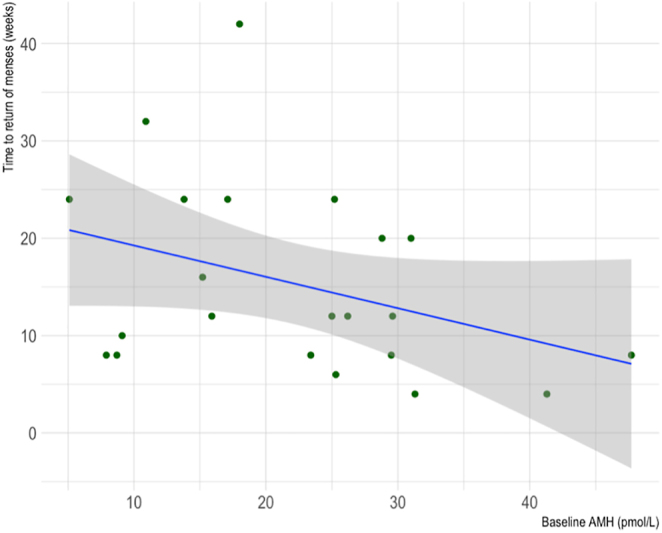
Scatter plot of the correlation between baseline serum AMH and time to return of menses for breast cancer patients who underwent AC. *R* = −0.38, *P* = 0.09.

As shown in Fig. 9, there was no statistically significant relationship between baseline serum AMH and time to return of menses for AC breast cancer patients (Spearman’s rank correlation coefficient = −0.38, *P* = 0.09).

**Figure 10:**
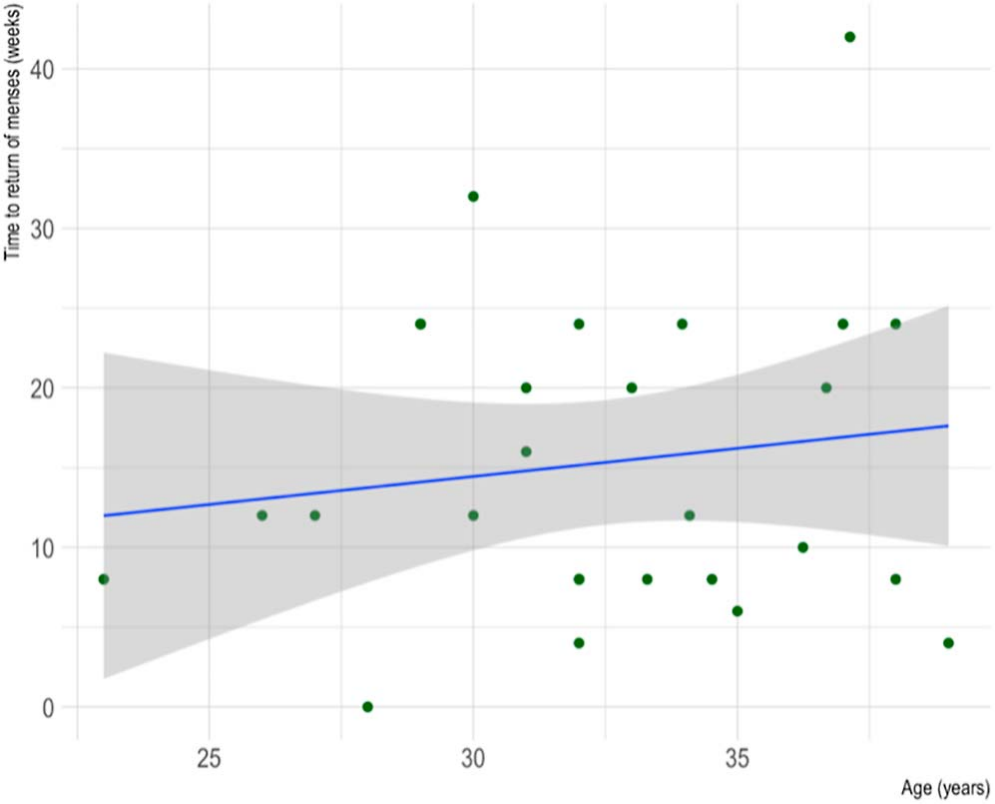
Scatter plot of the correlation between age and time to return of menses for breast cancer patients who underwent AC. *R* = 0.01, *P* = 0.97.

The relationship between time to return of menses and age for AC breast cancer patients, as displayed in Fig. 10, was also not statistically significant (Spearman’s rank correlation coefficient = 0.01, *P* = 0.97).

These outcomes for pairwise correlation of time to return of menses and variables are summarised in Table 3.

**Table 3:** Outcomes for pairwise correlation (Spearman’s rank correlation coefficient (r_s_)) between time to return of menses and variables.

Variable	r_s_	*P*
All patients		
Baseline AMH (pmol/L)	−0.14	0.36
Age (years)	−0.03	0.83
Alkylating (all patients)		
Baseline AMH (pmol/L)	−0.40	0.03
Age (years)	0.12	0.44
Non-alkylating (all patients)		
Baseline AMH (pmol/L)	−0.07	0.82
Age (years)	−0.36	0.13
Alkylating (breast cancer)		
Baseline AMH (pmol/L)	−0.38	0.09
Age (years)	0.01	0.97

Analysis of breast cancer patients who underwent NAC was not performed due to the low sample size (*n* = 5).

### Changes in AMH levels at 6 and 12 months post-completion of chemotherapy

**Figure 11:**
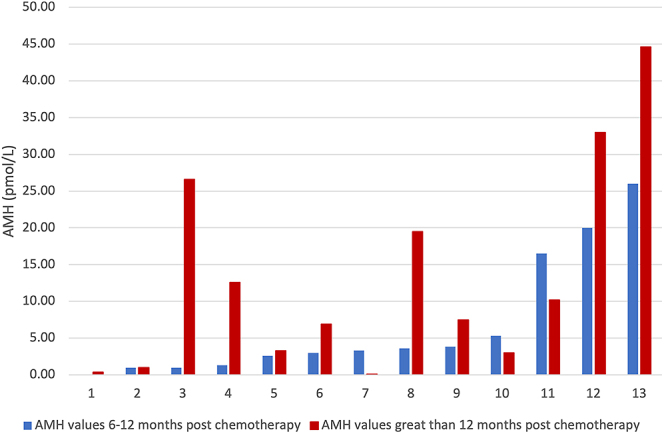
A comparison of AMH serum levels in patients at 6–12 months and more than 12 months after chemotherapy.

AMH values 6 months after completion were recorded for 21 patients. The mean (±SD) AMH 6 months post-chemotherapy was 7.44 ± 7.94 pmol/L. AMH values ranged from 0 to 26.00 pmol/L. AMH values 12 months after completion of chemotherapy were recorded for 27 patients. The mean AMH 12 months post-chemotherapy was 14.43 (±13.27) pmol/L, ranging from 0.11 to 44.60 pmol/L. As shown in Fig. 11, when comparing AMH serum levels for patients for whom data were obtained at both 6–12 months and more than 12 months after chemotherapy, 9/13 (69.23%) patients had a rise in AMH levels over time, while 3/13 (23.08%) had a decrease and 1/13 (0.77%) remained the same. Only 2/13 patients were not taking goserelin for chemoprotection. One patient had a decrease in their AMH level between 6 and 12 months. The other patient had an increase in their AMH level between 6 and 12 months.

## Discussion

The findings of this retrospective study suggest that the duration of return of menses is related to pre-chemotherapy ovarian reserve as measured by serum AMH levels in patients treated with AC agents. Evidence for the predictive ability of AMH when it comes to the duration of return of menses is limited in the literature. A prospective study by Su *et al*. found that higher pre-chemotherapy AMH levels (greater than 0.7 ng/mL) were associated with an earlier resumption of ovarian function (measured by menses) in breast cancer patients undergoing chemotherapy (Su *et al.* 2014). The role of AMH in the setting of chemotherapy was also explored by Anderson *et al.* (2013), who found pre-chemotherapy AMH to be the only reliable biomarker in assessing the likelihood of CRA. We observed that women with higher AMH levels prior to AC had an earlier return of menses. This also suggested that serum AMH reflects ovarian activity for this cohort, and CRA may indicate ovarian function, as has been suggested by other groups (Anderson & Cameron 2011). These findings are concordant with the conclusions of two groups (Anderson & Cameron 2011, Xue *et al.* 2019) that serum AMH determined at the time of diagnosis is predictive of long-term ovarian function (5 years) after chemotherapy. A lower pre-chemotherapy AMH was found to be associated with higher rates of CRA at 6-month follow-up (Loubersac *et al.* 2020). This is useful in clinical counselling of cancer patients as it could allow for better individual predictions of outcomes after AC and hence more tailored strategies for fertility preservation. We were unable to demonstrate a statistically significant correlation between pre-chemotherapy AMH and time to return of menses for the NAC cohort or for the breast cancer patients who underwent AC cohort; however, this may be due to the limited sample size (*n* = 15 and *n* = 23, respectively), as this analysis in both groups yielded a negative correlation.

This study corroborates the lack of association between age at the beginning of chemotherapy and time to return of menses for patients who have undergone both AC and NAC, as has previously been described in the literature. Findings of other groups demonstrated that AMH was the only predictor of long-term ovarian function after chemotherapy, superior to both age and follicle-stimulating hormone (FSH) (Anderson & Cameron 2011). Other studies have supported that FSH is of little clinical utility for prediction due to its significant variation during cycles and the unfeasibility of early follicular phase blood tests (Su *et al.* 2014). It is known that the follicular pool declines with advancing age; therefore, age is a marker of the primordial follicle pool. However, it is also known that there is great variation in ovarian reserve within and between different age groups (de Kat *et al.* 2016). The study conducted by Su *et al*. found younger age (less than 40 years old) to be associated with an earlier resumption of menses in breast cancer patients following AC (Su *et al.* 2014); however, the median age for this cohort was 39.5, with an IQR from 36.4 to 42.9. This is a significantly older and more limited cohort than our study, with a median age of 32 and a range of 18–40 years old for patients who underwent AC and therefore did not incorporate younger patients with a potentially higher baseline ovarian reserve.

We endeavoured to examine whether ovarian reserve can recover after chemotherapy. A study by Decanter and coworkers found that in a group of 32 women below the age of 40 diagnosed with breast cancer, AMH levels progressively increased during the first year after the completion of AC. This coincided with a resumption of menstruation (Decanter *et al.* 2018). In addition, Decanter and coworkers found a complete recovery of AMH levels to pre-chemotherapy levels within 6 months for patients who had undergone NAC (Decanter *et al.* 2010). A study by Brougham and coworkers consisting mostly of pre-pubertal girls (77%) found AMH remained undetectable even at 3 years in patients undergoing a chemotherapy regimen deemed to have high gonadotoxic risk (determined by chemotherapy drug, duration and in some cases radiotherapy to ovaries) (Brougham *et al.* 2012). From our cohort of patients who resumed menses, we observed that there was an increase in AMH serum levels from the period of 6 months after chemotherapy to a point in time more than 12 months after chemotherapy in 69.23% of patients. However, for the other 30.77%, AMH levels either decreased or remained static. Goserelin could not be assessed as a contributing factor in this study, with 76.92% of patients taking goserelin for the duration of their chemotherapy. A study by Dillon and coworkers involving breast cancer (41%) and lymphoma patients (37%), 72% of whom received AC, observed recovery of AMH 9 months after chemotherapy and also found a greater recovery rate in patients with higher baseline AMH values (Dillon *et al.* 2013).

This study has several strengths. First, it answers the question of which factors correlate with the duration of return of menses, which, to our knowledge, has not been assessed before. The median time for the return of menses was found to be 12 weeks for all patients. This allows for the provision of more individualised information to patients as to what to expect following the conclusion of chemotherapy. We were able to demonstrate statistical significance for the correlation between baseline AMH and time to return of menses for cancer patients treated with AC. Second, the cohort is population-based and included women with different types of cancers, with a subgroup analysis of breast cancer patients. This makes it easier to extrapolate the results to patient demographics seen in fertility preservation settings. It may help clinicians decide the timing of fertility preservation strategies and thus help minimise the long-term impacts of chemotherapy on women of reproductive age.

A significant limitation of this study is that the data were collected retrospectively. Therefore, not all patients had AMH serum levels assessed both at 6 and 12 months after the completion of chemotherapy. We propose that future prospective studies should record AMH serum levels at 6 and 12 months, as well as dosage and duration of chemotherapy in their subjects to allow for the variation in AMH levels between individual patients (de Kat *et al.* 2016) and chemotherapy regimens. Furthermore, the number of treatment cycles and dosage of chemotherapy are known to influence the recovery of AMH (Anderson *et al.* 2022). As this retrospective study was carried out in a gynaecological outpatient setting without access to oncological patient files, data on dosage and duration of chemotherapy were unable to be obtained. This study is also limited by its sample size, perhaps contributing to the lack of statistically significant results seen in the subgroup analysis of breast cancer patients. The self-reporting of time to return of menses could potentially result in reporting bias. In addition, women who were given prolonged gonadotropin-releasing hormone (GnRH) agonist treatment for breast cancer after chemotherapy could not be included in the group due to medically induced amenorrhoea. Jacobson *et al*. demonstrated that women who were diagnosed with cancer after the age of 30 were more likely to experience irregular periods after temporary amenorrhoea, possibly a manifestation of damage to the ovaries or perimenopause (Jacobson *et al.* 2016). Several studies (Zervoudis *et al.* 2010, Letourneau *et al.* 2012) explore the prevalence of infertility in women after the completion of chemotherapy despite a return of menses. The predictive value of AMH when it comes to fecundability is still unclear in the literature (Peigné & Decanter 2014). However, this was not assessed in our patient cohort due to the retrospective nature of this study.

Further research into the recovery of AMH following chemotherapy is required to assist in reassuring patients when confronted with low AMH levels in the early stages after chemotherapy. In addition, due to the lack of longer-term follow-up in most studies conducted, better knowledge about AMH recovery after chemotherapy would allow for considerations of the need for contraception in the period after chemotherapy, the commencement time for neoadjuvant hormone treatments in breast cancer and the ability to perform post-chemotherapy oocyte retrieval in the case of poor retrieval prior to chemotherapy. Measuring serum AMH levels at 6 and 12 months after treatment and yearly for 5 years may be a valuable part of the follow-up of cancer patients in a fertility preservation service.

The issue of female fertility preservation following cancer treatments has recently become increasingly prominent with improving survival rates. Pre-chemotherapy AMH can be recommended as a biological marker of ovarian reserve and was found to correlate with return of menses for women with all types of cancer following completion of AC. Age was not shown to be a reliable determinant of the duration of return of menses. This information can be used by clinicians for counselling and individualisation of fertility preservation strategies. Larger prospective studies are required to find AMH levels for prediction of time to return of menses and assess the longer-term recovery of AMH after chemotherapy, fecundability and the influence of individual chemotherapy regimens and the use of GnRH agonists for ovarian protection.

## Declaration of interest

The authors declare that there is no conflict of interest that could be perceived as prejudicing the impartiality of the research reported. A Polyakov is an Associate Editor of *Reproduction & Fertility* and was not involved in the review or editorial process for this paper on which he is listed as an author.

## Funding

This research did not receive any specific grant from funding agencies in the public, commercial or not-for-profit sectors.

## Author contributions

MS, RK, FA and KS contributed to the conception and design of the study. Data were acquired by MS, RK, FA and AL. The data were analysed and interpreted by MS, AL and AP. The manuscript was drafted by MS and RK. The manuscript was revised by FA, AL, SMA, AP, GR and KS.
